# Socioeconomic Differences in Patient Reported Outcome Measures 3 Months After Stroke: A Nationwide Swedish Register-Based Study

**DOI:** 10.1161/STROKEAHA.124.047172

**Published:** 2024-07-01

**Authors:** Anita Lindmark, Mia von Euler, Eva-Lotta Glader, Katharina S. Sunnerhagen, Marie Eriksson

**Affiliations:** Department of Statistics, Umeå School of Business, Economics and Statistics (A.L., M.E.), Umeå University, Sweden.; Department of Public Health and Clinical Medicine (E.-L.G.), Umeå University, Sweden.; School of Medicine, Department of Neurology and Rehabilitation, Örebro University, Sweden (M.v.E.).; Institute of Neuroscience and Physiology, Rehabilitation Medicine, Sahlgrenska Academy, University of Gothenburg, and the Sahlgrenska University Hospital, Sweden (K.S.S.).

**Keywords:** activities of daily living, health status, low socioeconomic status, Patient Reported Outcome Measures, stroke

## Abstract

**BACKGROUND:**

There is a well-known association between low socioeconomic status (SES), poor survival, and clinician-reported outcomes after stroke. We aimed to assess socioeconomic differences in Patient Reported Outcome Measures 3 months after stroke.

**METHODS:**

This nationwide cohort study included patients registered with acute stroke in the Swedish Stroke Register 2015–2017. Patient Reported Outcome Measures included activities of daily living (mobility, toileting, and dressing), and poststroke symptoms (low mood, fatigue, pain, and poor general health). Information on SES prestroke was retrieved from Statistics Sweden and defined by a composite measure based on education and income tertiles. Associations between SES and Patient Reported Outcome Measures were analyzed using logistic regression adjusting for confounders (sex and age) and additionally for potential mediators (stroke type, severity, cardiovascular disease risk factors, and living alone). Subgroup analyses were performed for stroke type, men and women, and younger and older patients.

**RESULTS:**

The study included 44 511 patients. Of these, 31.1% required assistance with mobility, 18% with toileting, and 22.2% with dressing 3 months after stroke. For poststroke symptoms, 12.3% reported low mood, 39.1% fatigue, and 22.7% pain often/constantly, while 21.4% rated their general health as poor/very poor. Adjusted for confounders, the odds of needing assistance with activities of daily living were highest for patients with low income and primary school education, for example, for mobility, odds ratio was 2.06 (95% CI, 1.89–2.24) compared with patients with high income and university education. For poststroke symptoms, odds of poor outcome were highest for patients with low income and university education (eg, odds ratio, 1.79 [95% CI, 1.49–2.15] for low mood). Adjustments for potential mediators attenuated but did not remove associations. The associations were similar in ischemic and hemorrhagic strokes and more pronounced in men and patients <65 years old.

**CONCLUSIONS:**

There are substantial SES-related differences in Patient Reported Outcome Measures poststroke. The more severe outcome associated with low SES is more pronounced in men and in patients of working age.

There are social inequalities in access to stroke care and outcome.^1,2^ Previous studies on the relationship between low socioeconomic status (SES) and more severe prognoses have mainly focused on survival and clinician-reported outcomes. Patient Reported Outcome Measures (PROMs) represent the patient’s self-perceived health, as reported directly by the patient without interpretation by others. Typically, it includes measures of functional status, symptom burden, and health-related quality of life. There is an increasing interest in the use of PROMs in stroke care and research to improve patient outcomes and quality of care and to reduce inequalities in health.^3^

To date, only a few studies have considered the relationship between SES and PROMs after stroke. An observational cohort study of 1195 ischemic stroke patients in a US cerebrovascular clinic 2015 to 2017 showed that lower household income was associated with worse PROMs in several domains, including anxiety, depression, pain, sleep disturbance, executive function, physical function, and satisfaction with social roles.^4,5^ In Brazil, 175 patients at a good-quality public stroke center were less likely to be able to walk, communicate, manage toilets and clothing, and had a reduced quality of life 90 days poststroke compared with 165 patients in a private hospital with the same stroke protocol.^6^ Additional studies, based on physicians’ assessments of patient questionnaires or interviews, report inconsistent results. A community-based, prospective study from Kolkata, India, including 241 patients 2006 to 2010, reported an association between low income, low education, and poststroke depression.^7^ A cohort study, including 556 patients from the PRAISE study (Prevent Recurrent All-Inner City Stroke Through Education) conducted in New York City between 2009 and 2012, identified ethnic differences in the risk of poststroke depression but no significant differences related to income.^8^ The NEMESIS (North East Melbourne Stroke Incidence Study), including 225 first strokes, showed that low SES (defined by the occupations of the patient and spouse) was associated with worse health-related quality of life at 2 years poststroke.^9^ The mechanisms by which SES affects PROMs are unclear, and effect modifiers and mediating factors (eg, related to lifestyle) have not been studied.

Using a nationwide register-based cohort from the Swedish Stroke Register (Riksstroke), we aimed to quantify socioeconomic differences in PROMs 3 months after stroke and assess how potential mediating and modifying factors influence differences. By using a 2-dimensional measure of SES, we further aimed to evaluate how level of education and income interact and affect PROMs in men and women of working age and older age.

## METHODS

### Materials

Because of the sensitive nature of the data collected for this study, requests to access the data set from qualified researchers trained in human subject confidentiality protocols may be sent to Riksstroke at riksstroke@regionvasterbotten.se, subject to ethical approval and permission from Statistics Sweden.

**Figure 1.**
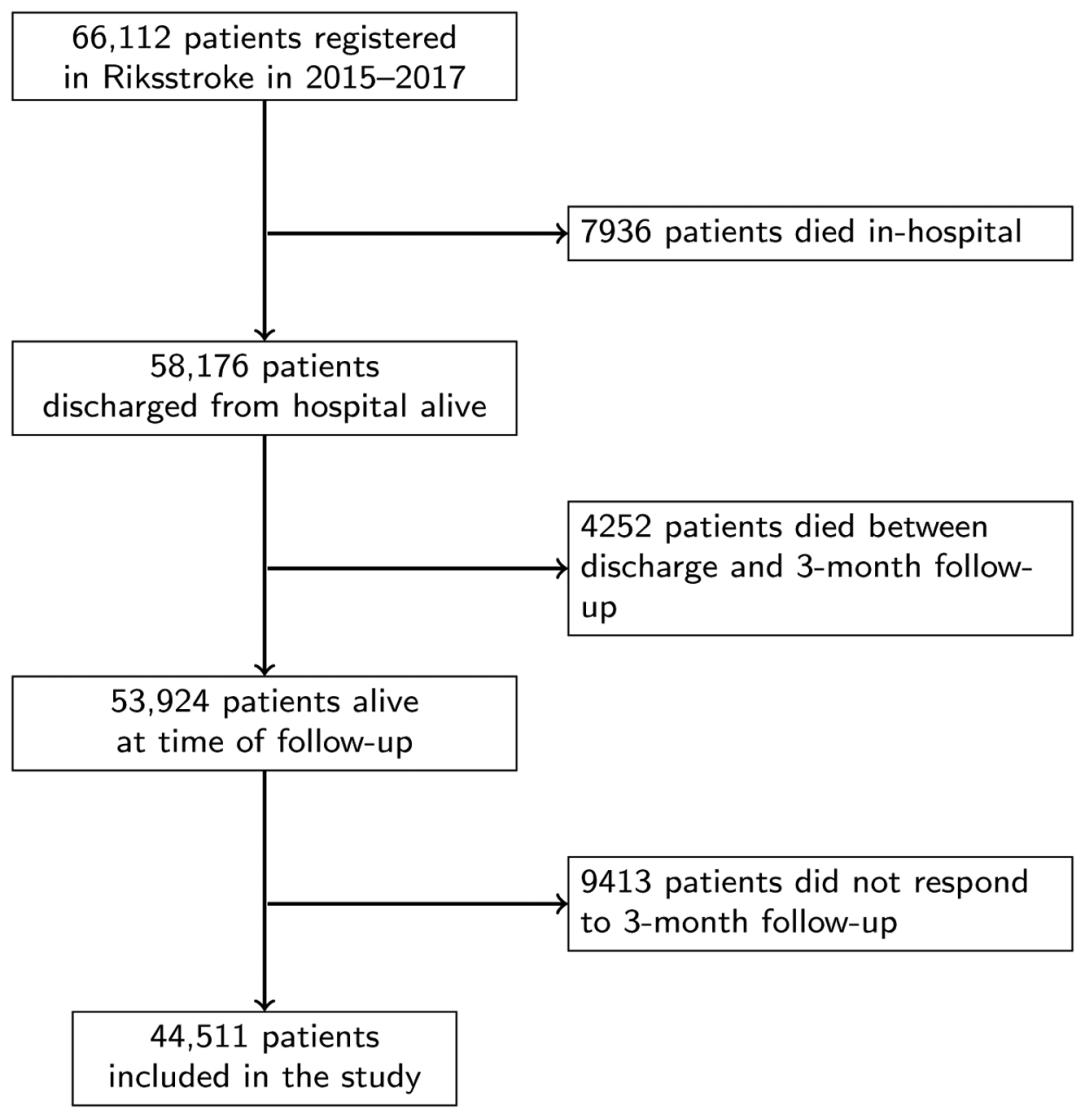
**Study flow chart.** Riksstroke indicates Swedish Stroke Register.

Riksstroke collects patient-level data from all 72 Swedish hospitals providing acute stroke care. The aim of the register is to monitor and support improvements in stroke care quality, and data are collected both at the acute stage (registered by hospital staff) and at follow-up 3 and 12 months after stroke (through questionnaires administered by the hospitals but filled in by the patients).^10^ Riksstroke covers over 90% of all acute stroke patients in Sweden,^11^ and in 2017, around 85% of patients registered at the acute stage were followed up or deceased at the 3-month follow-up.^12^ All patients registered with an acute stroke in Riksstroke in 2015 to 2017 and who provided follow-up information at 3 months were eligible for inclusion in the current study (see Figure 1 for the study flow chart).

Information on socioeconomic factors was retrieved by linking Riksstroke data with data from the LISA (Longitudinal Integrated Database for Health Insurance and Labor Market Studies), managed by Statistics Sweden. The linkage was performed at the patient level using Swedish national identification numbers. Data were linked by Statistics Sweden, and pseudonymized data were returned to the researchers.

This study adheres to the RECORD (Reporting of Studies Conducted Using Observational Routinely-Collected Health Data) guidelines.^13^

### Variables

The outcome variables were PROMs at the Riksstroke 3-month follow-up and included variables related to activities of daily living (ADL) and poststroke symptoms. The ADL variables were mobility (independent indoors and outdoors/independent indoors, assisted outdoors/assisted indoors and outdoors), toileting (completely independent/needs assistance), and dressing (completely independent/needs assistance). The poststroke symptoms were experience of low mood, fatigue, and pain (all rated on the scale never or almost never/sometimes/often/constantly/do not know), as well as self-rated general health (very good/quite good/quite poor/very poor/do not know). The simple PROM questions used in Riksstroke have been validated against established instruments.^14^

For the main analyses, the PROMs were dichotomized as poor outcome versus good outcome. For the ADL-related variables, poor outcome was defined, respectively, as needing assistance moving around outdoors or both indoors and outdoors, needing assistance toileting, and needing assistance dressing. For the poststroke symptoms, a poor outcome was defined as experiencing low mood, fatigue, and pain, respectively, often/constantly, and rating their general health as quite poor/very poor. Patients answering “do not know” were treated as missing.

The exposure SES was defined by a composite measure of the highest attained education level (primary school/secondary school/university) and income level. Income level was based on the individual’s portion of the family’s disposable income 2 years before the stroke. The individual’s portion of the family’s disposable income is constructed to consider the size and composition (number of adults and children) of the household and estimate the portion of the total household income that is disposed of by each individual.^15^ It is derived by taking the sum of the incomes minus taxes of all family members in the household, multiplied by the consumption weight (meant to reflect the consumption needs) of the individual, and divided by the total consumption weight of the family. The income levels were defined by year-specific tertiles for the income of all patients registered in Riksstroke in 2015 to 2017, with low income defined as the lowest tertile, high income as the highest tertile, and mid-income everyone in between. SES was categorized as the 9 combinations of primary school/secondary school/university and low/mid/high income.

Baseline confounders were sex (male/female) and patient age at the time of stroke. Potential mediating variables, all measured before or at the time of stroke, were stroke subtype, level of consciousness based on the reaction level scale (RLS; 1=fully conscious, RLS 2–3=lucid but drowsy, RLS 4–8=unconscious), dependence in ADL before stroke, smoking status (smoker versus nonsmoker or unknown), diabetes, atrial fibrillation, previous stroke, and whether the patient was living alone. The confounders and potential mediators were ascertained from hospital charts and registered in Riksstroke by hospital staff.

### Statistical Methods

Patient characteristics for the whole study population as well as separated by SES are presented as frequencies and proportions for categorical variables and as median and interquartile range for continuous variables.

Unadjusted associations between SES and PROMs were analyzed through proportions with 95% CIs. The associations between SES and PROMs adjusted for (1) baseline covariates (sex, age, and age-squared) and (2) baseline covariates and potential mediators were analyzed through separate logistic regression models for each PROM. The results are presented as estimated odds ratios (ORs) with a 95% CI.

To investigate the differences in the associations between SES and PROMs between ischemic and hemorrhagic strokes, men and women, as well as between older and younger patients, interaction terms were added to the model. Subsequently, subgroup analyses were performed. The age categories contrasted were patients aged ≤64 years and patients aged ≥65 years. The choice of 65 years as the age cutoff was motivated by this being the standard age of retirement in Sweden during the study period.

We performed complete case analyses, meaning that for each analysis, patients missing information on any of the variables of interest in that analysis were excluded.

All analyses were performed using R version 4.2.2.^16^

### Ethical Considerations

This research is covered by ethical approval from the regional ethics review board in Umeå, Sweden (reference number 2017/184-31). Information regarding their inclusion in the Riksstroke registry, as well as the registry’s objectives, is provided to patients and their next of kin. They are also given the option to choose not to participate (opt-out consent).

## RESULTS

### Study Population

A total of 53 924 stroke patients were alive at the time of the 3-month follow-up and therefore eligible for the study (Figure 1). Of these, 44 511 patients (82.5%) who were followed up and had data on 1 or more of the PROMs were included in the study. The 9413 patients who did not respond to the follow-up questionnaire (Figure 1) were more likely to have low SES, lowered consciousness at baseline, hemorrhagic stroke, and have had a previous stroke (Table S1). They were also more likely to be ADL-dependent at baseline, to smoke, to have diabetes, and to be living alone. The median age in the study population was 76 years (interquartile range, 68–83), and 45.3% were female (Table).

**Table.**
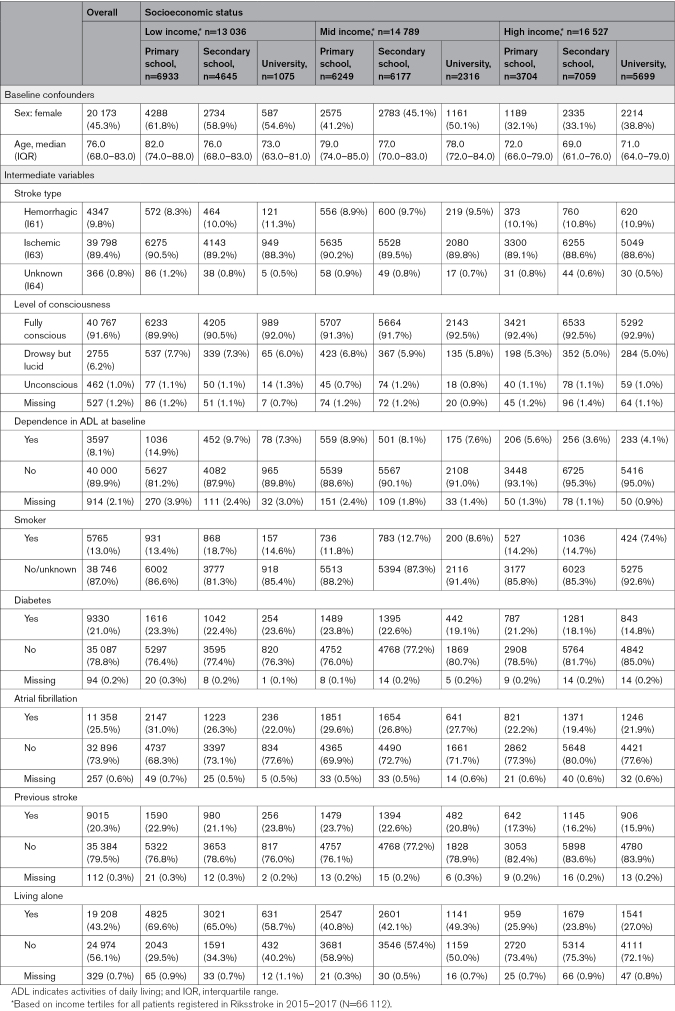
Patient Characteristics, Number (%) for the Study Population (n=44 511) and Separated by Socioeconomic Status

### Socioeconomic Status

Overall, 29.3% of patients were classified as having low, 33.2% as having mid, and 37.1% as having high income, while 37.9% had only primary school education, 40.2% had secondary school, and 20.4% had university education. The proportion of patients with only primary school education was 53.2% in low-income patients, 42.2% in mid-income patients, and 22.4% in high-income patients. The corresponding proportions of university education were 8.2% in low-income patients, 15.7% in mid-income patients, and 34.5% in high-income patients.

The proportion of female patients decreased with increasing income levels. Among patients with low income, the proportion of female patients also decreased with increasing education level, while the opposite was observed among patients with mid or high income (Table). Patients with low income and only primary school education were the oldest on average, while patients with high income were the youngest.

For the possible mediators, the SES differences in stroke type and level of consciousness were small (Table). High-income patients were less likely to be dependent in ADL at baseline, to have had a previous stroke, to have diabetes, to have atrial fibrillation, and to be living alone. Patients with secondary school education had the highest proportion of smokers within each income level.

### Patient Reported Outcome Measures

A large proportion of patients reported ADL dependency or poststroke symptoms (Table S2). Overall, 31.1% (95% CI, 30.7–31.5) of patients required assistance with mobility outdoors or both indoors and outdoors; 18% (95% CI, 17.7–18.4) required assistance with toileting; and 22.2% (95% CI, 21.8–22.6) with dressing. For the poststroke symptom measures, 12.3% (95% CI, 12.0–12.6) of patients reported having low mood often/constantly, while 39.1% (95% CI, 38.6–39.5) reported experiencing fatigue and 22.7% (95% CI, 22.3–23.1) pain often/constantly, and 21.4% (95% CI, 21.1–21.8) of patients rated their general health as quite poor/very poor.

### Missing Values

In the study population, 0.3% of patients were missing information on income and 1.5% on education level. The proportions of missing values on the potential mediators ranged from 0.2% to 2.1%, with the largest proportion for ADL dependency at baseline (Table). The proportions missing patient characteristics were generally similar across SES levels, with the exception of ADL dependency at baseline where lower SES levels had higher proportions missing (Table).

Among responders to the follow-up questionnaire, the proportion of missing values on ADL variables ranged from 1.0% to 1.5%, and for poststroke symptoms (where “do not know” was classified as missing), the proportion ranged between 3.7% and 7.3%, with the largest proportion for general health (Table S2). Differences in proportions missing between SES levels were small for ADL variables, while patients with lower SES tended to have larger proportions missing poststroke symptoms.

### PROMs and SES

**Figure 2.**
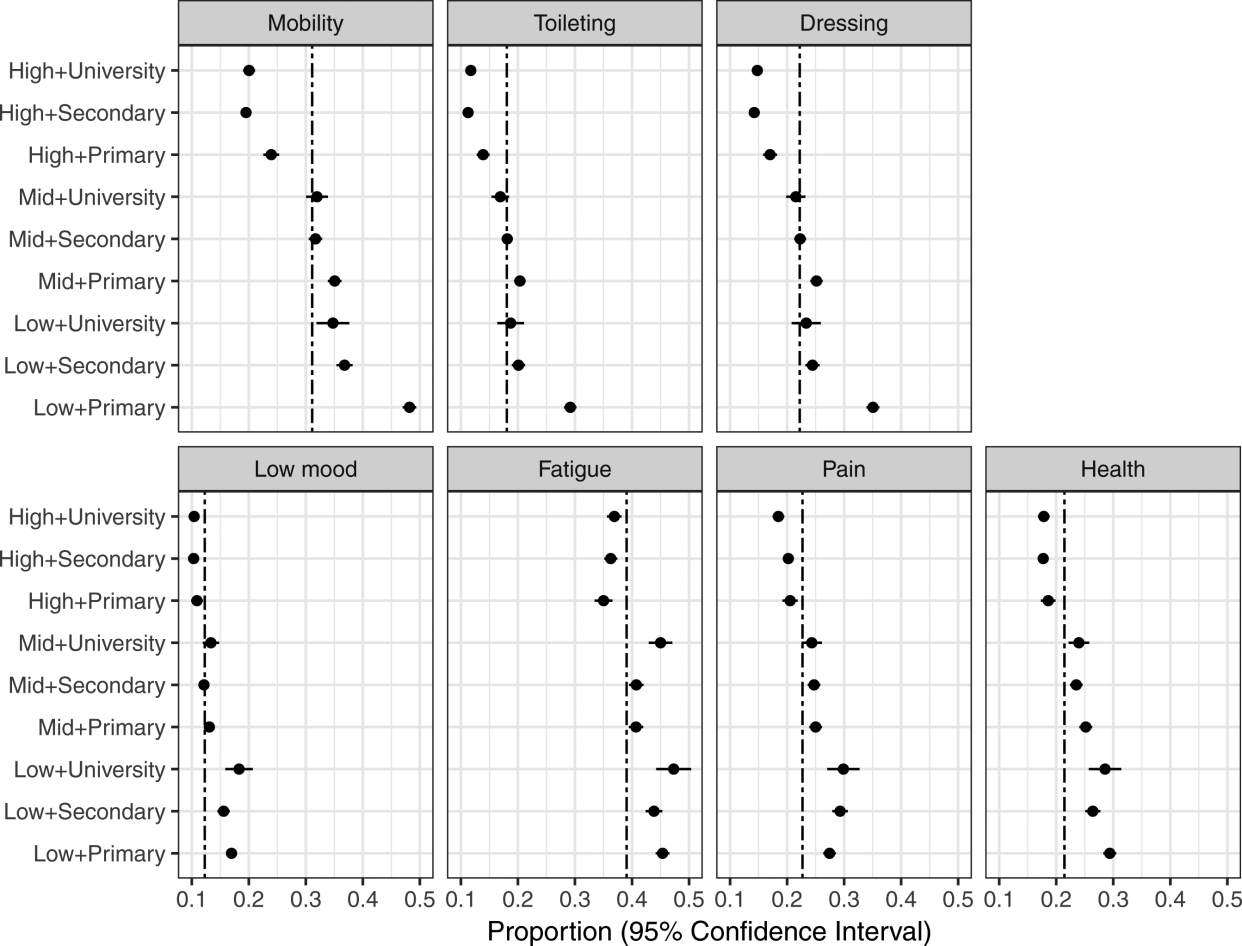
**Proportions (with 95% CIs) of patients reporting poor outcome separated by socioeconomic status.** The double-dash lines correspond to the overall proportion for each patient reported outcome.

The proportions that needed assistance with ADL (mobility, toileting, and dressing; Figure 2) were highest for patients with both low income and only primary school education and generally lower for patients with high income, regardless of education level.

For the poststroke symptoms (low mood, fatigue, pain, and general health; Figure 2), there was an income gradient, with increasing proportions of patients with poor outcomes for decreasing income levels. Within income levels, the association between education and poor outcomes varied. University-educated patients had the highest proportions of fatigue within all income groups, while patients with only primary school education had the highest proportions of poor general health in all income groups. For low mood and pain, the within-income level educational differences were less clear-cut, although the largest proportion of low mood was observed in the group with low income and university education (18.3% [95% CI, 15.9%–20.7%]).

**Figure 3.**
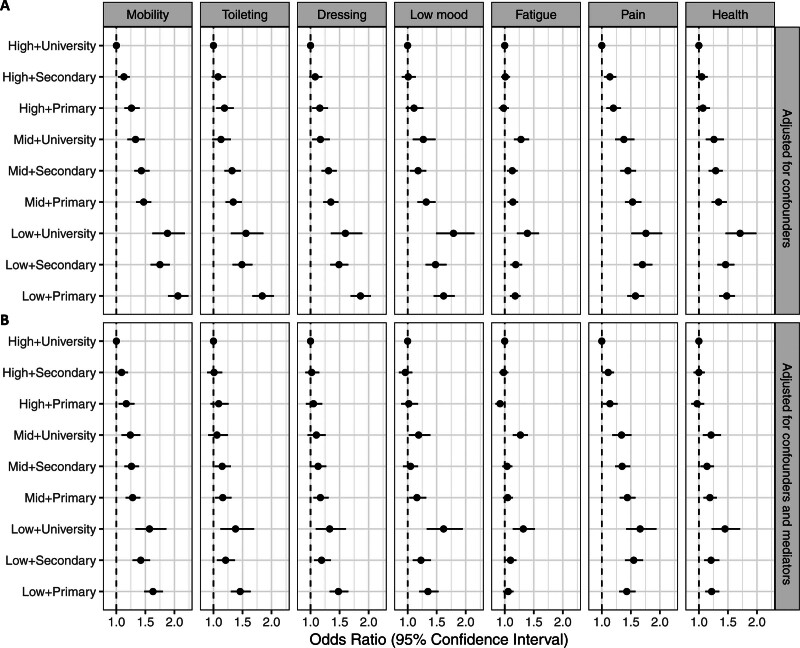
**Odds ratios with 95% CIs for socioeconomic status (income tertile+highest attained education) from logistic regression models with patient reported poor outcome measures as outcomes. A**, Adjusted for sex, age, and age-squared. **B**, Adjusted for sex, age, age-squared, stroke type, level of consciousness, activities of daily living at baseline, smoking status, diabetes, atrial fibrillation, previous stroke, and living alone.

Overall, the associations between low SES and more severe outcomes remained after adjustments for differences in sex and age. Patients with high income and university education were less likely to experience poor outcomes for all PROMs (Figure 3A; Table S3). For all 3 ADL variables, the group with low income and only primary school had the highest odds of poor outcome compared with the group with high income and university education, with estimated ORs of 2.06 (95% CI, 1.89–2.24) for mobility, 1.84 (95% CI, 1.67–2.04) for toileting, and 1.86 (95% CI, 1.69–2.04) for dressing.

For poststroke symptoms, the group with low income and university education stood out with the most increased odds of poor outcome compared with the group with high income and university education. The differences were of a similar magnitude for low mood, pain, and general health, with estimated ORs of 1.79 (95% CI, 1.49–2.15), 1.76 (95% CI, 1.51–2.05), and 1.71 (95% CI, 1.46–2.00), but smaller for fatigue, with an estimated OR of 1.39 (95% CI, 1.21–1.59).

After additional adjustments for differences in the potential mediators (stroke type, level of consciousness, ADL at baseline, known smoker, diabetes, atrial fibrillation, previous stroke, and living alone), the same general patterns remained, but the associations were attenuated (Figure 3B; Table S3).

### Subgroup Analyses of Stroke Type, Sex, and Age

There was little evidence for differences in the associations between SES and PROMs between patients with ischemic and hemorrhagic stroke (*P* values for stroke type [ischemic or hemorrhagic stroke]-by-SES interaction >0.10 for all PROMs except toileting; Figure S1).

**Figure 4.**
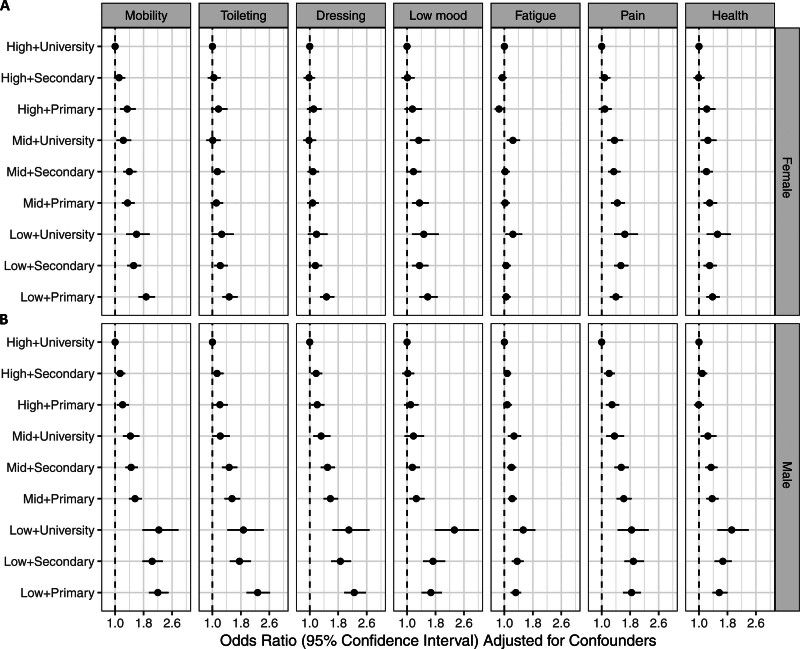
**Odds ratios with 95% CIs for socioeconomic status (income tertile+highest attained education) from logistic regression models with patient reported poor outcome measures as outcomes adjusted for sex, age, and age-squared.** Among (**A**) female and (**B**) male patients.

Overall, there was a SES gradient in both men and women, and the age-adjusted negative associations between lower SES and PROMs were even more pronounced in men compared with women (*P* values for sex-by-SES interaction <0.05 for all PROMs except pain; Table S4). In particular, lower income appeared to be more strongly associated with poor outcomes in men than in women. (Figure 4; Table S4). In women, the highest estimated ORs of poor outcome compared with the highest SES group (high income and university education) ranged from 1.24 to 1.87 for the different PROMs after adjustments for age. In men, the corresponding estimated ORs ranged from 1.53 to 2.33 (Table S4). With additional adjustment for potential mediators, similar patterns were observed with attenuated associations (Figure S2; Table S4).

**Figure 5.**
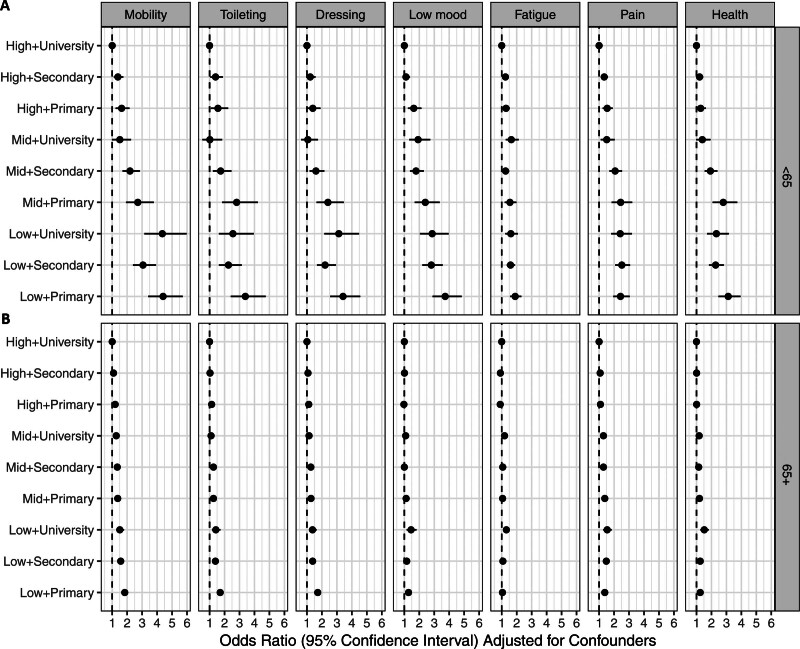
**Odds ratios with 95% CIs for socioeconomic status (income tertile+highest attained education) from logistic regression models with patient reported poor outcome measures as outcomes adjusted for sex, age, and age-squared.** Among (**A**) patients aged <65 years and (**B**) patients aged 65 years and above (65+).

The associations between low SES and poor outcomes were much more pronounced in patients <65 years old compared with older patients (*P* values for age category-by-SES interaction <0.001 for all PROMs; Table S5). The highest estimated ORs, compared with the highest SES group, ranged from 1.90 to 4.40 in patients aged <65 years compared with 1.28 to 1.84 in older patients (Figure 5; Table S5) after adjustments for sex. These patterns remained with additional adjustment for potential mediators (Figure S3; Table S5).

## DISCUSSION

This nationwide study showed that patients with lower income and education were much more likely to experience and report a less favorable outcome in all domains (mobility, dressing, toileting, low mood, fatigue, pain, and perceived health). A part of this association appeared to be mediated by differences in stroke characteristics, patient living situation, functional status, or cardiovascular comorbidities. A substantial part of the income-related associations remained, while the association with education was modest after adjustments for age, sex, and mediating factors. The socioeconomic gradient was more pronounced in men and in patients of working age, that is, younger than 65 years.

We observed SES-related differences in PROMs that for several domains were even higher than the 10% absolute increased risk of death or dependency that has recently been reported between ischemic stroke patients with low versus high income and education in Sweden^17^ and for several measures of SES worldwide.^2^ The overall association between income and PROMs seen in our study is in agreement with that observed for physical and executive function, pain, fatigue, anxiety, and depression in a cohort including stroke patients from the Cleveland Clinic.^5^ However, that study did not report age- or sex-specific disparities.

We used a composite SES measure, based on education and income, to better capture different aspects of SES.^18^ While education is commonly set early in life, income may vary over the course of life.^19^ The observed sex gap, with a higher proportion of women in the low SES groups, is likely related to age. The level of education has increased in Sweden, and women are on average older than men at the time of stroke. In addition, women tend to have older spouses, which affects their family income. This could partly explain the different associations between SES and PROMs in men and women. Also, the income span is reduced after retirement, which is likely to partly explain why the association was attenuated in patients aged ≥65 years.

Somewhat unexpectedly, patients with university education and low income reported a similar or worse outcome compared with patients with lower education and the same income level. We had no information on the source of income, and a potential explanation could be related to comorbidities and sick leave.

The study results are based on nationwide register data covering a large population but were limited to patients who responded to the 3-month follow-up questionnaire. The response rate was generally high (>80%) and the proportion of missing data among the responders was low, but it cannot be ruled out that the associations between SES and PROMs could have been different if all patients had responded. Comparing the patient characteristics of responders and nonresponders, we found an overrepresentation of low SES, CVD risk factors, hemorrhagic stroke, ADL dependency, and lowered consciousness at baseline among nonresponders. This could indicate that patients who did not respond did so due to a poorer outcome and that the SES differences observed could be even larger. The study results are generalizable to the Swedish context but may not be applicable outside of this.

Another limitation of the study is that the PROMs were not based on established instruments. To increase the response rate of the Riksstroke follow-up, the number of questions has been kept at a minimum, and the PROM questions are therefore simplified. However, the PROMs used in Riksstroke have been validated against established instruments (Barthel Index, Beck Depression Inventory, Fatigue Symptom Inventory, Brief Pain Inventory Short Form, and SF-12).^20^ In general, the specificity was high for all Riksstroke PROMs, ranging from 75% (fatigue) to 100% (health). The sensitivity was also high for ADL, pain, and fatigue (95%–98%), while it was modest for health and low mood (24%–38%).

There were mediating factors that we did not include, and as in all observational studies, we cannot exclude unobserved confounding. We did not have access to information about, for example, stroke volume and location, presence of coagulopathy or large vessel occlusion, and ischemic stroke subtype. We had limited information on comorbidities, and level of consciousness based on RLS was used as a proxy for stroke severity. An alternative measure could be the NIHSS; however, the proportion of missing values in the study population was >40%. As a sensitivity analysis, we imputed the missing NIHSS, and adjusting for this imputed variable instead of level of consciousness gave very similar results (not shown) and required making untestable assumptions regarding the missingness mechanism. Therefore, we opted to use the more complete RLS-based variable. We do not correct for multiple testing in our analyses but have been careful to focus on general patterns rather than highlight isolated statistically significant results. Our results demonstrated an association between low SES and poor outcome, which should not be interpreted as a causal effect.

The main aim of the current study was to investigate SES differences in PROMs with a secondary aim to identify possible mediating factors. Future studies focusing specifically on explaining the mechanisms behind the SES-PROM relationships are of interest.

## CONCLUSIONS

Patients with a combination of low education and low income are more prone to experience and report a more severe outcome 3 months after stroke than other patient groups. The SES-related differences can only in part be explained by differences in age, sex, and mediating factors, including stroke type and severity, and are more pronounced in men and in patients of working age. The socioeconomic gradient varies between education and income measures, which demonstrates the importance of considering different aspects of SES in studies of SES-related inequalities.

## ARTICLE INFORMATION

### Acknowledgments

The authors are grateful to Riksstroke and all the participating hospitals.

### Sources of Funding

The study was funded by FORTE (Swedish Research Council for Health, Working Life and Welfare, grant 2018-00852) and Vetenskapsrådet (Swedish Research Council, grant 2018-02670).

### Disclosures

Dr von Euler is the chair of Riksstroke. The other authors report no conflicts.

### Supplemental Material

Figures S1–S3

Tables S1–S5

## Supplementary Material


