# Supplementary material for: Socioeconomic Differences in Patient Reported Outcome Measures 3 Months After Stroke: A Nationwide Swedish Register-Based Study
Source: Stroke. 2024 Jul 1;55(8):2055–65. doi: 10.1161/STROKEAHA.124.047172 (PMC11259239; doi:10.1161/STROKEAHA.124.047172)
Supplement: Supplementary file 1 [file str-55-2055-s001.pdf]

Supplementary material

Table S1: Patient characteristics for patients in the study population and patients who did not respond to the 3 month follow up.

	**Study population (44,511)**	**Non-responders (9,413)**
Socioeconomic status		
**High income+university**	5699 (12.8%)	916 (9.7%)
**High income+secondary**	7059 (15.9%)	1197 (12.7%)
**High income+primary**	3704 (8.3%)	592 (6.3%)
**Mid income+university**	2316 (5.2%)	440 (4.7%)
**Mid income+secondary**	6177 (13.9%)	1163 (12.4%)
**Mid income+primary**	6249 (14.0%)	1172 (12.5%)
**Low income+university**	1075 (2.4%)	354 (3.8%)
**Low income+secondary**	4645 (10.4%)	1305 (13.9%)
**Low income+primary**	6933 (15.6%)	1862 (19.8%)
**Missing**	654 (1.5%)	412 (4.4%)
Baseline confounders		
**Sex: Female**	20,173 (45.3%)	4370 (46.4%)
**Age, Median (IQR)**	76.0 (68.0, 83.0)	75.0 (64.0, 84.0)
Intermediate variables		
**Stroke type**		
**Hemorrhagic (I61)**	4347 (9.8%)	1250 (13.3%)
**Ischemic (I63)**	39,798 (89.4%)	8067 (85.7%)
**Unknown (I64)**	366 (0.8%)	96 (1.0%)
**Level of consciousness**		
**Fully conscious**	40,767 (91.6%)	8083 (85.9%)
**Drowsy but lucid**	2755 (6.2%)	895 (9.5%)
**Unconscious**	462 (1.0%)	196 (2.1%)
**Missing**	527 (1.2%)	239 (2.5%)
**Dependence in ADL at baseline**		
**Yes**	3597 (8.1%)	1389 (14.8%)
**No**	40,000 (89.9%)	7564 (80.4%)
**Missing**	914 (2.1%)	460 (4.9%)

**Smoker**		
**Yes**	5765 (13.0%)	1584 (16.8%)
**No/Unknown**	38,746 (87.0%)	7829 (83.2%)
**Diabetes**		
**Yes**	9330 (21.0%)	2200 (23.4%)
**No**	35,087 (78.8%)	7119 (75.6%)
**Missing**	94 (0.2%)	94 (1.0%)
**Atrial fibrillation**		
**Yes**	11,358 (25.5%)	2514 (26.7%)
**No**	32,896 (73.9%)	6763 (71.8%)
**Missing**	257 (0.6%)	136 (1.4%)
**Previous stroke**		
**Yes**	9015 (20.3%)	2323 (24.7%)
**No**	35,384 (79.5%)	6985 (74.2%)
**Missing**	112 (0.3%)	105 (1.1%)
**Living alone**		
**Yes**	19,208 (43.2%)	4984 (52.9%)
**No**	24,974 (56.1%)	4215 (44.8%)
**Missing**	329 (0.7%)	214 (2.3%)

Table S2: Patient reported outcome measures, number (%), for the study population (n=44,511) and separated by socioeconomic status.

	**Overall**	**Socioeconomic status**								
		Low income[a] (n=13,036)			***Mid income[a] (n=14,789)***			***High income[a] (n=16,527)***		
		Primary school (n=6933)	***Secondary school (n=4645)***	***University (n=1075)***	***Primary school (n=6249)***	***Secondary school (n=6177)***	***University (n=2316)***	***Primary school (n=3704)***	***Secondary school (n=7059)***	***University (n=5699)***
**Mobility**										
**Independent indoors/outdoors**	30,000 (67.4%)	3527 (50.9%)	2881 (62.0%)	689 (64.1%)	3995 (63.9%)	4152 (67.2%)	1556 (67.2%)	2783 (75.1%)	5614 (79.5%)	4502 (79.0%)
**Independent indoors assisted outdoors**	7046 (15.8%)	1600 (23.1%)	890 (19.2%)	195 (18.1%)	1100 (17.6%)	1010 (16.4%)	395 (17.1%)	419 (11.3%)	705 (10.0%)	574 (10.1%)
**Assisted indoors/outdoors**	6799 (15.3%)	1680 (24.2%)	787 (16.9%)	172 (16.0%)	1057 (16.9%)	917 (14.8%)	336 (14.5%)	457 (12.3%)	656 (9.3%)	555 (9.7%)
**Missing**	666 (1.5%)	126 (1.8%)	87 (1.9%)	19 (1.8%)	97 (1.6%)	98 (1.6%)	29 (1.3%)	45 (1.2%)	84 (1.2%)	68 (1.2%)
**Toileting**										
**Completely independent**	35,956 (80.8%)	4850 (70.0%)	3654 (78.7%)	864 (80.4%)	4921 (78.7%)	4992 (80.8%)	1906 (82.3%)	3153 (85.1%)	6202 (87.9%)	4981 (87.4%)
**Needs assistance**	8034 (18.0%)	1999 (28.8%)	919 (19.8%)	199 (18.5%)	1257 (20.1%)	1105 (17.9%)	388 (16.8%)	509 (13.7%)	787 (11.1%)	662 (11.6%)
**Missing**	521 (1.2%)	84 (1.2%)	72 (1.6%)	12 (1.1%)	71 (1.1%)	80 (1.3%)	22 (1.0%)	42 (1.1%)	70 (1.0%)	56 (1.0%)
**Dressing**										
**Completely independent**	34,159 (76.7%)	4450 (64.2%)	3457 (74.4%)	817 (76.0%)	4631 (74.1%)	4744 (76.8%)	1804 (77.9%)	3044 (82.2%)	6004 (85.1%)	4815 (84.5%)
**Needs assistance**	9886 (22.2%)	2403 (34.7%)	1119 (24.1%)	249 (23.2%)	1557 (24.9%)	1361 (22.0%)	495 (21.4%)	624 (16.8%)	997 (14.1%)	835 (14.7%)
**Missing**	466 (1.0%)	80 (1.2%)	69 (1.5%)	9 (0.8%)	61 (1.0%)	72 (1.2%)	17 (0.7%)	36 (1.0%)	58 (0.8%)	49 (0.9%)

**Low mood**										
**Never/almost never**	18,373 (41.3%)	2263 (32.6%)	1681 (36.2%)	388 (36.1%)	2428 (38.9%)	2472 (40.0%)	946 (40.8%)	1776 (47.9%)	3413 (48.3%)	2816 (49.4%)
**Sometimes**	17,767 (39.9%)	2927 (42.2%)	1936 (41.7%)	425 (39.5%)	2596 (41.5%)	2600 (42.1%)	948 (40.9%)	1350 (36.4%)	2673 (37.9%)	2085 (36.6%)
**Often**	4235 (9.5%)	827 (11.9%)	522 (11.2%)	134 (12.5%)	590 (9.4%)	543 (8.8%)	224 (9.7%)	292 (7.9%)	550 (7.8%)	449 (7.9%)
**Constantly**	1231 (2.8%)	234 (3.4%)	146 (3.1%)	48 (4.5%)	166 (2.7%)	158 (2.6%)	68 (2.9%)	91 (2.5%)	151 (2.1%)	121 (2.1%)
**Do not know**	1666 (3.7%)	436 (6.3%)	205 (4.4%)	41 (3.8%)	277 (4.4%)	225 (3.6%)	72 (3.1%)	104 (2.8%)	138 (2.0%)	111 (1.9%)
**Missing**	1239 (2.8%)	246 (3.5%)	155 (3.3%)	39 (3.6%)	192 (3.1%)	179 (2.9%)	58 (2.5%)	91 (2.5%)	134 (1.9%)	117 (2.1%)
**Fatigue**										
**Never/almost never**	5639 (12.7%)	681 (9.8%)	527 (11.3%)	109 (10.1%)	810 (13.0%)	699 (11.3%)	268 (11.6%)	601 (16.2%)	1055 (14.9%)	824 (14.5%)
**Sometimes**	19,824 (44.5%)	2915 (42.0%)	1955 (42.1%)	429 (39.9%)	2742 (43.9%)	2817 (45.6%)	961 (41.5%)	1748 (47.2%)	3324 (47.1%)	2691 (47.2%)
**Often**	13,075 (29.4%)	2135 (30.8%)	1400 (30.1%)	345 (32.1%)	1768 (28.3%)	1852 (30.0%)	767 (33.1%)	969 (26.2%)	1975 (28.0%)	1662 (29.2%)
**Constantly**	4307 (9.7%)	851 (12.3%)	539 (11.6%)	138 (12.8%)	670 (10.7%)	567 (9.2%)	239 (10.3%)	297 (8.0%)	517 (7.3%)	394 (6.9%)
**Do not know**	642 (1.4%)	163 (2.4%)	95 (2.0%)	16 (1.5%)	107 (1.7%)	86 (1.4%)	24 (1.0%)	26 (0.7%)	67 (0.9%)	37 (0.6%)
**Missing**	1024 (2.3%)	188 (2.7%)	129 (2.8%)	38 (3.5%)	152 (2.4%)	156 (2.5%)	57 (2.5%)	63 (1.7%)	121 (1.7%)	91 (1.6%)
**Pain**										
**Never/almost never**	18,891 (42.4%)	2409 (34.7%)	1671 (36.0%)	401 (37.3%)	2502 (40.0%)	2519 (40.8%)	963 (41.6%)	1726 (46.6%)	3480 (49.3%)	3017 (52.9%)
**Sometimes**	13,122 (29.5%)	2246 (32.4%)	1389 (29.9%)	308 (28.7%)	1884 (30.1%)	1883 (30.5%)	708 (30.6%)	1092 (29.5%)	1954 (27.7%)	1476 (25.9%)
**Often**	6379 (14.3%)	1052 (15.2%)	773 (16.6%)	195 (18.1%)	903 (14.5%)	939 (15.2%)	342 (14.8%)	473 (12.8%)	887 (12.6%)	677 (11.9%)
**Constantly**	3725 (8.4%)	709 (10.2%)	495 (10.7%)	107 (10.0%)	560 (9.0%)	507 (8.2%)	195 (8.4%)	255 (6.9%)	490 (6.9%)	341 (6.0%)

**Do not know**	1056 (2.4%)	268 (3.9%)	153 (3.3%)	19 (1.8%)	179 (2.9%)	117 (1.9%)	46 (2.0%)	68 (1.8%)	100 (1.4%)	72 (1.3%)
**Missing**	1338 (3.0%)	249 (3.6%)	164 (3.5%)	45 (4.2%)	221 (3.5%)	212 (3.4%)	62 (2.7%)	90 (2.4%)	148 (2.1%)	116 (2.0%)
**General health**										
**Very good**	5015 (11.3%)	467 (6.7%)	400 (8.6%)	108 (10.0%)	621 (9.9%)	620 (10.0%)	239 (10.3%)	543 (14.7%)	1027 (14.5%)	945 (16.6%)
**Quite good**	26,662 (59.9%)	3912 (56.4%)	2704 (58.2%)	590 (54.9%)	3685 (59.0%)	3754 (60.8%)	1398 (60.4%)	2307 (62.3%)	4492 (63.6%)	3506 (61.5%)
**Quite poor**	7486 (16.8%)	1383 (19.9%)	862 (18.6%)	227 (21.1%)	1101 (17.6%)	1094 (17.7%)	412 (17.8%)	507 (13.7%)	979 (13.9%)	768 (13.5%)
**Very poor**	2056 (4.6%)	440 (6.3%)	252 (5.4%)	52 (4.8%)	348 (5.6%)	251 (4.1%)	104 (4.5%)	144 (3.9%)	209 (3.0%)	197 (3.5%)
**Do not know**	1846 (4.1%)	455 (6.6%)	249 (5.4%)	42 (3.9%)	289 (4.6%)	246 (4.0%)	74 (3.2%)	112 (3.0%)	196 (2.8%)	141 (2.5%)
**Missing**	1446 (3.2%)	276 (4.0%)	178 (3.8%)	56 (5.2%)	205 (3.3%)	212 (3.4%)	89 (3.8%)	91 (2.5%)	156 (2.2%)	142 (2.5%)

[a] Based on income tertiles for all patients registered in Riksstroke (n=66,112).

Table S3: Odds ratios (OR) with 95 % confidence intervals (CI) for socioeconomic status (SES) from logistic regression models with patient reported outcome measures as outcomes.

	**Adjusted for sex, age and age squared**			**Adjusted for sex, age, age squared, stroke type, level of consciousness, ADL at baseline, smoking status, diabetes, atrial fibrillation, previous stroke and living alone**		
	OR	*95% CI*	*P-value SES[a]*	*OR*	*95% CI*	*P-value SES[a]*
Mobility (Assisted outdoors or indoors/outdoors vs. independent indoors/outdoors)						
SES (ref. high income+university)	1		<0.001	1		<0.001
High income+secondary	1.13	1.03 – 1.23		1.09	0.98 – 1.20	
High income+primary	1.26	1.14 – 1.40		1.17	1.04 – 1.31	
Mid income+university	1.33	1.19 – 1.49		1.24	1.10 – 1.41	
Mid income+secondary	1.43	1.31 – 1.57		1.26	1.14 – 1.39	
Mid income+primary	1.47	1.34 – 1.60		1.28	1.16 – 1.41	
Low income+university	1.88	1.62 – 2.19		1.57	1.33 – 1.86	
Low income+secondary	1.75	1.59 – 1.92		1.42	1.28 – 1.58	
Low income+primary	2.06	1.89 – 2.24		1.63	1.48 – 1.80	
Toileting (Needs assistance vs. Completely independent)						
SES (ref. high income+university)	1		<0.001	1		<0.001
High income+secondary	1.08	0.97 – 1.21		1.01	0.89 – 1.15	
High income+primary	1.19	1.05 – 1.35		1.09	0.95 – 1.26	
Mid income+university	1.13	0.98 – 1.30		1.06	0.91 – 1.25	
Mid income+secondary	1.32	1.18 – 1.47		1.15	1.02 – 1.30	
Mid income+primary	1.34	1.21 – 1.49		1.16	1.03 – 1.31	
Low income+university	1.56	1.30 – 1.86		1.38	1.12 – 1.70	
Low income+secondary	1.49	1.33 – 1.67		1.21	1.06 – 1.37	
Low income+primary	1.84	1.67 – 2.04		1.46	1.30 – 1.64	
Dressing (Needs assistance vs. Completely independent)						
SES (ref. high income+university)	1		<0.001	1		<0.001
High income+secondary	1.08	0.98 – 1.20		1.02	0.91 – 1.15	
High income+primary	1.16	1.03 – 1.30		1.05	0.92 – 1.20	
Mid income+university	1.17	1.03 – 1.33		1.10	0.95 – 1.27	
Mid income+secondary	1.31	1.19 – 1.45		1.13	1.01 – 1.27	
Mid income+primary	1.35	1.22 – 1.48		1.17	1.05 – 1.31	
Low income+university	1.60	1.35 – 1.89		1.33	1.09 – 1.61	
Low income+secondary	1.49	1.34 – 1.65		1.19	1.06 – 1.35	

Low income+primary	1.86	1.69 – 2.04		1.48	1.33 – 1.65	
Low mood (Often/Constantly vs. Never/almost never/Sometimes)						
SES (ref. high income+university)	1		<0.001	1		<0.001
High income+secondary	1.01	0.90 – 1.14		0.96	0.85 – 1.08	
High income+primary	1.11	0.97 – 1.27		1.02	0.89 – 1.18	
Mid income+university	1.27	1.10 – 1.48		1.19	1.02 – 1.40	
Mid income+secondary	1.18	1.04 – 1.32		1.05	0.92 – 1.18	
Mid income+primary	1.32	1.17 – 1.48		1.16	1.03 – 1.32	
Low income+university	1.79	1.49 – 2.15		1.62	1.34 – 1.96	
Low income+secondary	1.48	1.31 – 1.67		1.23	1.09 – 1.40	
Low income+primary	1.62	1.44 – 1.81		1.35	1.20 – 1.53	
Fatigue (Often/Constantly vs. Never/almost never/Sometimes)						
SES (ref. high income+university)	1		<0.001	1		<0.001
High income+secondary	1.01	0.94 – 1.09		0.98	0.91 – 1.06	
High income+primary	0.98	0.90 – 1.07		0.92	0.84 – 1.01	
Mid income+university	1.28	1.16 – 1.42		1.27	1.14 – 1.40	
Mid income+secondary	1.13	1.04 – 1.22		1.04	0.96 – 1.13	
Mid income+primary	1.14	1.05 – 1.23		1.05	0.97 – 1.14	
Low income+university	1.39	1.21 – 1.59		1.32	1.14 – 1.52	
Low income+secondary	1.19	1.10 – 1.30		1.10	1.01 – 1.20	
Low income+primary	1.18	1.09 – 1.27		1.06	0.97 – 1.15	
Pain (Often/Constantly vs. Never/almost never/Sometimes)						
SES (ref. high income+university)	1		<0.001	1		<0.001
High income+secondary	1.14	1.04 – 1.25		1.11	1.01 – 1.21	
High income+primary	1.20	1.08 – 1.33		1.14	1.02 – 1.27	
Mid income+university	1.38	1.23 – 1.56		1.34	1.18 – 1.51	
Mid income+secondary	1.45	1.32 – 1.59		1.35	1.23 – 1.49	
Mid income+primary	1.53	1.40 – 1.68		1.44	1.31 – 1.58	
Low income+university	1.76	1.51 – 2.05		1.66	1.42 – 1.94	
Low income+secondary	1.70	1.55 – 1.87		1.55	1.40 – 1.71	
Low income+primary	1.58	1.44 – 1.73		1.43	1.30 – 1.58	
General health (Very poor/quite poor vs. very good/quite good)						
SES (ref. high income+university)	1		<0.001	1		<0.001
High income+secondary	1.05	0.95 – 1.15		1.00	0.91 – 1.10	
High income+primary	1.07	0.96 – 1.19		0.97	0.87 – 1.09	

Mid income+university	1.26	1.12 – 1.43		1.21	1.07 – 1.38	
Mid income+secondary	1.29	1.17 – 1.41		1.14	1.03 – 1.26	
Mid income+primary	1.34	1.22 – 1.48		1.19	1.08 – 1.31	
Low income+university	1.71	1.46 – 2.00		1.45	1.22 – 1.71	
Low income+secondary	1.46	1.32 – 1.61		1.21	1.09 – 1.35	
Low income+primary	1.48	1.35 – 1.62		1.22	1.11 – 1.35	

ADL: Activities of daily living.

[a] P-value from likelihood ratio test of outcome models with and without SES.

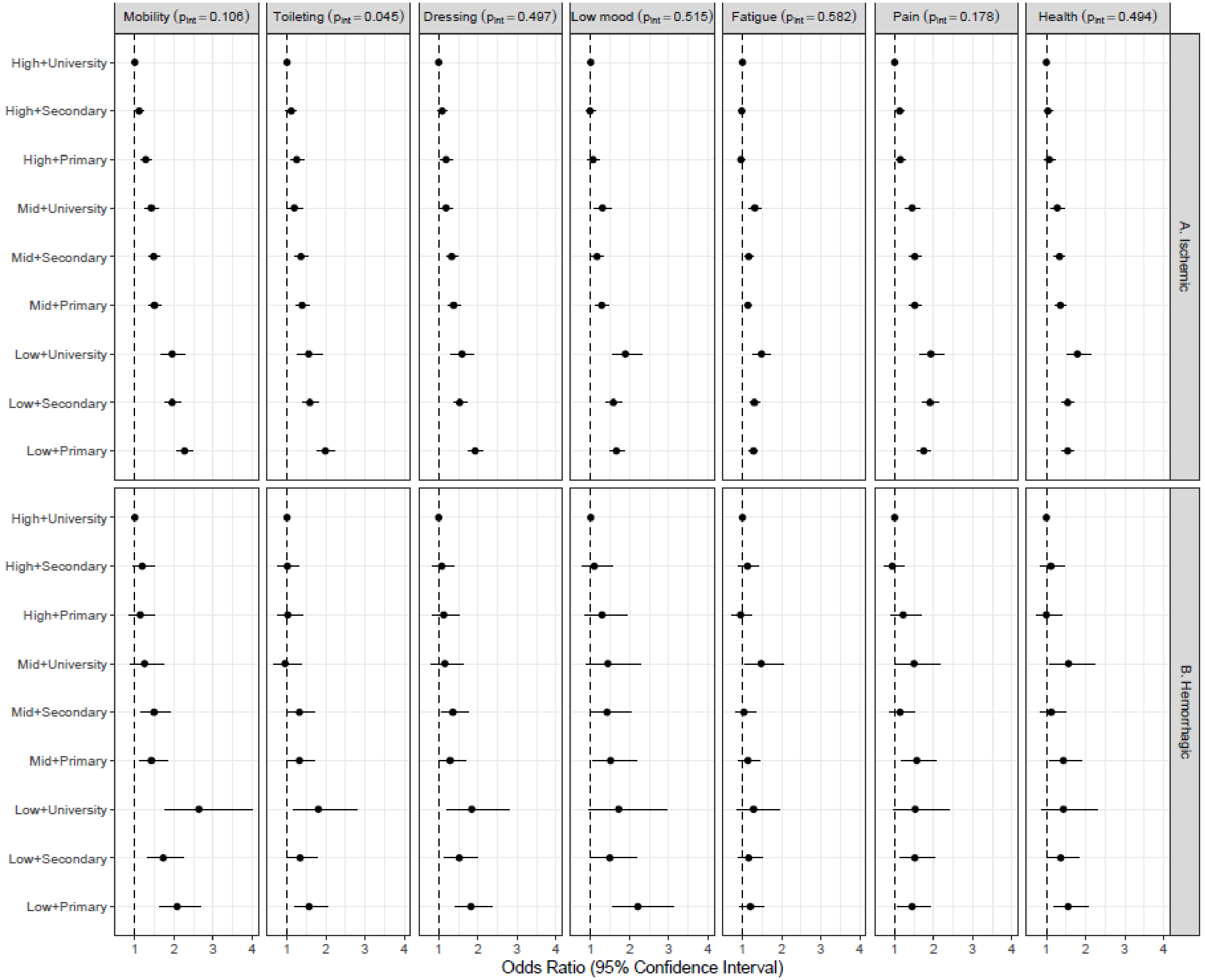

Figure S1: Odds ratios with 95 % confidence intervals for socioeconomic status from logistic regression models with poor patient reported outcome measures as outcomes adjusted for sex, age, and age squared. Among A. Ischemic and B. Hemorrhagic stroke patients. p_{int} denotes the p-value from a likelihood ratio test of the interaction between socioeconomic status and stroke type (ischemic or hemorrhagic stroke).

Table S4: Odds ratios (OR) with 95 % confidence intervals (CI) for socioeconomic status (SES) from logistic regression models with patient reported outcome measures as outcomes, separated by sex.

	**Adjusted for age and age squared**					**Adjusted for sex, age, age squared, stroke type, level of consciousness, ADL at baseline, smoking status, diabetes, atrial fibrillation, previous stroke and living alone**				
	**Female (n=20,173)**		**Male (n=24,338)**			**Female (n=20,173)**		**Male (n=24,338)**		
	OR (95% CI)	*P-value SES[a]*	*OR (95% CI)*	*P-value SES[a]*	*P-value sex*SES[b]*	*OR (95% CI)*	*P-value SES[a]*	*OR (95% CI)*	*P-value SES[a]*	*P-value sex*SES[b]*
Mobility (Assisted outdoors or indoors/outdoors vs. independent indoors/outdoors)										
SES (ref. high income+university)	1	<0.001	1	<0.001	0.011	1	<0.001	1	<0.001	0.120
High income+secondary	1.11 (0.97 – 1.28)		1.14 (1.01 – 1.28)			1.06 (0.90 – 1.23)		1.11 (0.97 – 1.27)		
High income+primary	1.34 (1.14 – 1.58)		1.21 (1.05 – 1.39)			1.15 (0.96 – 1.38)		1.17 (1.01 – 1.37)		
Mid income+university	1.23 (1.04 – 1.45)		1.43 (1.22 – 1.69)			1.17 (0.98 – 1.40)		1.32 (1.10 – 1.58)		
Mid income+secondary	1.40 (1.23 – 1.60)		1.45 (1.29 – 1.64)			1.25 (1.08 – 1.44)		1.25 (1.09 – 1.44)		
Mid income+primary	1.35 (1.19 – 1.55)		1.56 (1.39 – 1.75)			1.18 (1.02 – 1.36)		1.37 (1.20 – 1.56)		
Low income+university	1.60 (1.31 – 1.97)		2.23 (1.78 – 2.78)			1.36 (1.08 – 1.70)		1.88 (1.46 – 2.42)		
Low income+secondary	1.52 (1.34 – 1.73)		2.04 (1.77 – 2.34)			1.28 (1.11 – 1.48)		1.61 (1.38 – 1.88)		
Low income+primary	1.87 (1.66 – 2.12)		2.20 (1.95 – 2.50)			1.53 (1.33 – 1.75)		1.71 (1.49 – 1.98)		
Toileting (Needs assistance vs. Completely independent)										
SES (ref. high income+university)	1	<0.001	1	<0.001	0.007	1	0.300	1	<0.001	0.003
High income+secondary	1.04 (0.87 – 1.23)		1.13 (0.97 – 1.30)			0.94 (0.77 – 1.14)		1.09 (0.92 – 1.28)		

High income+primary	1.17<br>(0.96 – 1.42)		1.21<br>(1.02 – 1.43)			0.98<br>(0.78 – 1.22)		1.20<br>(0.99 – 1.44)		
Mid income+university	1.01<br>(0.83 – 1.23)		1.22<br>(1.00 – 1.49)			0.95<br>(0.76 – 1.19)		1.13<br>(0.90 – 1.42)		
Mid income+secondary	1.14<br>(0.97 – 1.34)		1.47<br>(1.27 – 1.70)			0.98<br>(0.82 – 1.17)		1.31<br>(1.11 – 1.54)		
Mid income+primary	1.11<br>(0.95 – 1.30)		1.55<br>(1.34 – 1.78)			0.93<br>(0.78 – 1.12)		1.38<br>(1.17 – 1.62)		
Low income+university	1.26<br>(0.99 – 1.61)		1.87<br>(1.43 – 2.45)			1.07<br>(0.81 – 1.41)		1.76<br>(1.29 – 2.40)		
Low income+secondary	1.22<br>(1.04 – 1.43)		1.76<br>(1.49 – 2.08)			1.00<br>(0.83 – 1.19)		1.39<br>(1.14 – 1.69)		
Low income+primary	1.47<br>(1.28 – 1.70)		2.27<br>(1.96 – 2.62)			1.12<br>(0.95 – 1.32)		1.92<br>(1.62 – 2.27)		
Dressing (Needs assistance vs. Completely independent)										
SES (ref. high income+university)	1	<0.001	1	<0.001	<0.001	1	0.005	1	<0.001	<0.001
High income+secondary	0.98<br>(0.83 – 1.15)		1.18<br>(1.03 – 1.35)			0.89<br>(0.74 – 1.07)		1.15<br>(0.99 – 1.34)		
High income+primary	1.11<br>(0.92 – 1.33)		1.21<br>(1.04 – 1.41)			0.92<br>(0.75 – 1.13)		1.18<br>(1.00 – 1.40)		
Mid income+university	0.98<br>(0.82 – 1.18)		1.32<br>(1.11 – 1.58)			0.93<br>(0.76 – 1.14)		1.22<br>(0.99 – 1.49)		
Mid income+secondary	1.09<br>(0.94 – 1.26)		1.50<br>(1.31 – 1.71)			0.92<br>(0.78 – 1.09)		1.31<br>(1.13 – 1.53)		
Mid income+primary	1.08<br>(0.93 – 1.25)		1.58<br>(1.39 – 1.79)			0.91<br>(0.77 – 1.07)		1.40<br>(1.21 – 1.63)		
Low income+university	1.19<br>(0.95 – 1.50)		2.10<br>(1.65 – 2.69)			0.95<br>(0.73 – 1.24)		1.83<br>(1.38 – 2.42)		
Low income+secondary	1.16<br>(1.01 – 1.35)		1.86<br>(1.60 – 2.16)			0.95<br>(0.80 – 1.12)		1.45<br>(1.22 – 1.74)		
Low income+primary	1.47<br>(1.29 – 1.69)		2.25<br>(1.97 – 2.58)			1.15<br>(0.99 – 1.35)		1.86<br>(1.59 – 2.18)		
Low mood (Often/Constantly vs. Never/almost never/Sometimes										

SES (ref. high income+university)	1	<0.001	1	<0.001	0.027	1	<0.001	1	<0.001	0.107
High income+secondary	1.01 (0.85 – 1.21)		1.02 (0.87 – 1.19)			0.94 (0.78 – 1.13)		0.99 (0.85 – 1.17)		
High income+primary	1.15 (0.93 – 1.42)		1.10 (0.92 – 1.32)			1.04 (0.83 – 1.29)		1.04 (0.86 – 1.25)		
Mid income+university	1.33 (1.08 – 1.63)		1.18 (0.94 – 1.48)			1.27 (1.03 – 1.57)		1.06 (0.83 – 1.34)		
Mid income+secondary	1.18 (1.00 – 1.40)		1.15 (0.97 – 1.36)			1.06 (0.89 – 1.26)		1.02 (0.85 – 1.21)		
Mid income+primary	1.35 (1.14 – 1.61)		1.26 (1.07 – 1.49)			1.19 (1.00 – 1.42)		1.12 (0.94 – 1.33)		
Low income+university	1.47 (1.14 – 1.90)		2.33 (1.79 – 3.03)			1.38 (1.07 – 1.80)		2.02 (1.53 – 2.67)		
Low income+secondary	1.35 (1.14 – 1.60)		1.73 (1.45 – 2.07)			1.16 (0.97 – 1.38)		1.40 (1.16 – 1.69)		
Low income+primary	1.58 (1.35 – 1.86)		1.67 (1.41 – 1.98)			1.38 (1.17 – 1.63)		1.32 (1.11 – 1.58)		
Fatigue (Often/Constantly vs. Never/almost never/Sometimes)										
SES (ref. high income+university)	1	<0.001	1	<0.001	0.006	1	<0.001	1	0.002	0.006
High income+secondary	0.94 (0.83 – 1.06)		1.08 (0.98 – 1.19)			0.92 (0.81 – 1.04)		1.04 (0.94 – 1.14)		
High income+primary	0.85 (0.73 – 0.98)		1.08 (0.97 – 1.21)			0.77 (0.66 – 0.90)		1.03 (0.92 – 1.16)		
Mid income+university	1.24 (1.07 – 1.44)		1.27 (1.10 – 1.47)			1.25 (1.07 – 1.45)		1.22 (1.06 – 1.42)		
Mid income+secondary	1.02 (0.91 – 1.15)		1.20 (1.08 – 1.33)			0.96 (0.85 – 1.08)		1.10 (0.99 – 1.23)		
Mid income+primary	1.02 (0.90 – 1.15)		1.22 (1.10 – 1.35)			0.96 (0.85 – 1.09)		1.12 (1.00 – 1.24)		
Low income+university	1.24 (1.03 – 1.50)		1.53 (1.25 – 1.87)			1.18 (0.97 – 1.43)		1.44 (1.17 – 1.77)		
Low income+secondary	1.05 (0.94 – 1.18)		1.36 (1.21 – 1.54)			0.99 (0.88 – 1.12)		1.21 (1.07 – 1.38)		

Low income+primary	1.05 (0.94 – 1.18)		1.32 (1.18 – 1.47)			0.95 (0.85 – 1.07)		1.16 (1.03 – 1.30)		
Pain (Often/Constantly vs. Never/almost never/Sometimes)										
SES (ref. high income+university)	1	<0.001	1	<0.001	0.163	1	<0.001	1	<0.001	0.304
High income+secondary	1.08 (0.94 – 1.24)		1.21 (1.07 – 1.36)			1.04 (0.91 – 1.20)		1.17 (1.03 – 1.33)		
High income+primary	1.09 (0.92 – 1.28)		1.29 (1.12 – 1.49)			1.02 (0.86 – 1.21)		1.25 (1.08 – 1.44)		
Mid income+university	1.36 (1.16 – 1.60)		1.36 (1.13 – 1.63)			1.30 (1.10 – 1.53)		1.34 (1.11 – 1.61)		
Mid income+secondary	1.34 (1.18 – 1.53)		1.55 (1.36 – 1.76)			1.25 (1.09 – 1.43)		1.45 (1.27 – 1.66)		
Mid income+primary	1.44 (1.26 – 1.64)		1.62 (1.42 – 1.84)			1.35 (1.18 – 1.55)		1.52 (1.33 – 1.74)		
Low income+university	1.65 (1.35 – 2.02)		1.84 (1.45 – 2.32)			1.55 (1.26 – 1.91)		1.75 (1.38 – 2.23)		
Low income+secondary	1.54 (1.35 – 1.75)		1.89 (1.64 – 2.19)			1.41 (1.23 – 1.62)		1.69 (1.46 – 1.97)		
Low income+primary	1.40 (1.23 – 1.58)		1.84 (1.60 – 2.10)			1.28 (1.13 – 1.46)		1.64 (1.42 – 1.89)		
General health (Very poor/quite poor vs. very good/quite good)										
SES (ref. high income+university)	1	<0.001	1	<0.001	0.008	1	0.006	1	<0.001	0.097
High income+secondary	0.99 (0.85 – 1.15)		1.09 (0.96 – 1.23)			0.92 (0.79 – 1.08)		1.05 (0.93 – 1.19)		
High income+primary	1.22 (1.03 – 1.46)		0.99 (0.86 – 1.14)			1.06 (0.88 – 1.27)		0.93 (0.80 – 1.08)		
Mid income+university	1.25 (1.05 – 1.49)		1.25 (1.05 – 1.49)			1.21 (1.01 – 1.46)		1.18 (0.99 – 1.42)		
Mid income+secondary	1.21 (1.05 – 1.39)		1.34 (1.18 – 1.52)			1.08 (0.93 – 1.25)		1.17 (1.03 – 1.34)		
Mid income+primary	1.30 (1.13 – 1.50)		1.37 (1.21 – 1.55)			1.18 (1.02 – 1.37)		1.18 (1.03 – 1.34)		

Low income+university	1.52<br>(1.22 – 1.89)		1.92<br>(1.52 – 2.41)			1.34<br>(1.07 – 1.69)		1.56<br>(1.22 – 1.99)		
Low income+secondary	1.30<br>(1.13 – 1.49)		1.67<br>(1.44 – 1.92)			1.12<br>(0.97 – 1.30)		1.33<br>(1.14 – 1.55)		
Low income+primary	1.38<br>(1.21 – 1.58)		1.57<br>(1.38 – 1.80)			1.18<br>(1.03 – 1.36)		1.25<br>(1.08 – 1.44)		

[a] P-value from likelihood ratio test of outcome models with and without SES.

[b] P-value from likelihood ratio test of outcome models fitted on the whole study population with and without the SES*sex interaction

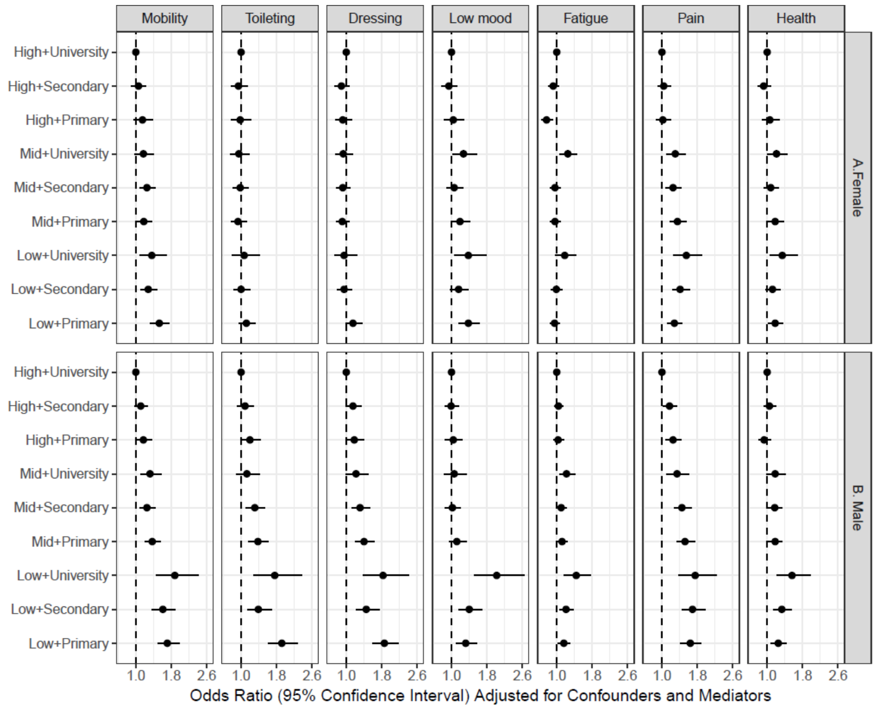

Figure S2: Odds ratios with 95 % confidence intervals for socioeconomic status from logistic regression models with poor patient reported outcome measures as outcomes adjusted for sex, age, age squared, stroke type, level of consciousness, ADL at baseline, smoking status, diabetes, atrial fibrillation, previous stroke and living alone. Among A. Female and B. Male patients.

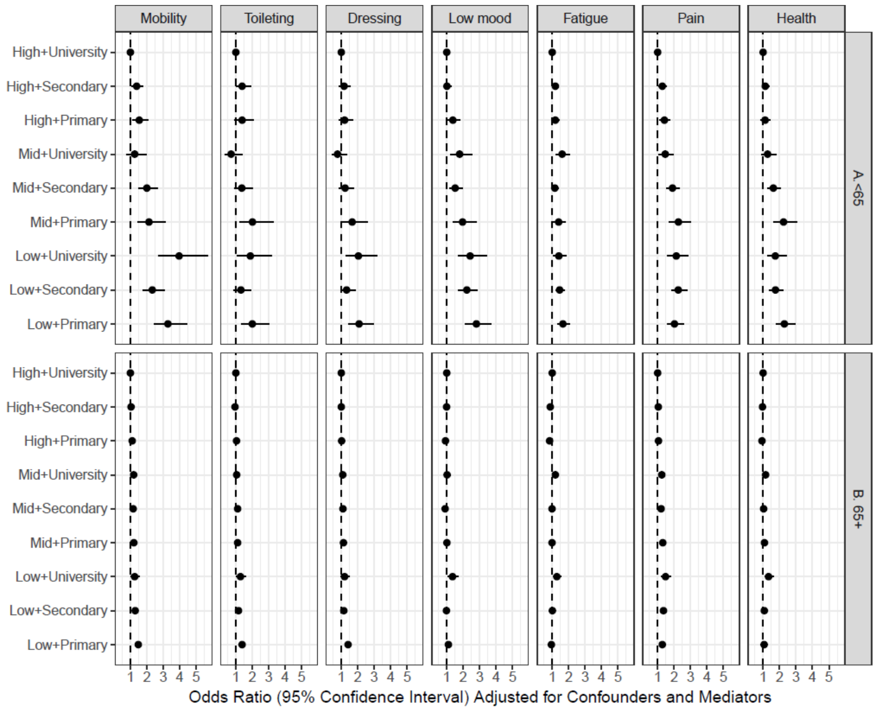

Figure S3: Odds ratios with 95 % confidence intervals for socioeconomic status from logistic regression models with poor patient reported outcome measures as outcomes adjusted for sex, age, age squared, stroke type, level of consciousness, ADL at baseline, smoking status, diabetes, atrial fibrillation, previous stroke and living alone. Among A. Patients aged <65 years, and B. Patients aged 65 years and above.

Table S5: Odds ratios (OR) with 95 % confidence intervals (CI) for socioeconomic status (SES) from logistic regression models with patient reported outcome measures as outcomes, separated by age categories (<65 and 65+ years).

	**Adjusted for sex, age and age squared**					**Adjusted for sex, age, age squared, stroke type, level of consciousness, ADL at baseline, smoking status, diabetes, atrial fibrillation, previous stroke and living alone**				
	**<65 (n=8157)**		**65+ (n=36,354)**			**<65 (n=8157)**		**65+ (n=36,354)**		
	OR (95%CI)	*P-value SES*[a]	*OR (95%CI)*	*P-value SES*[a]	*P-value SES*age65*[b]	*OR (95%CI)*	*P-value SES*[a]	*OR (95%CI)*	*P-value SES*[a]	*P-value SES*age65*[b]
Mobility (Assisted outdoors or indoors/outdoors vs. independent indoors/outdoors)										
SES (ref. high income+university)	1	<0.001	1	<0.001	<0.001	1	<0.001	1	<0.001	<0.001
High income+secondary	1.38 (1.10 – 1.73)		1.09 (0.98 – 1.21)			1.38 (1.08 – 1.77)		1.04 (0.93 – 1.17)		
High income+primary	1.63 (1.23 – 2.16)		1.20 (1.07 – 1.35)			1.54 (1.13 – 2.10)		1.11 (0.98 – 1.26)		
Mid income+university	1.51 (0.99 – 2.29)		1.27 (1.13 – 1.43)			1.26 (0.79 – 2.02)		1.20 (1.05 – 1.37)		
Mid income+secondary	2.19 (1.69 – 2.85)		1.34 (1.21 – 1.47)			2.00 (1.49 – 2.68)		1.17 (1.06 – 1.30)		
Mid income+primary	2.71 (1.93 – 3.79)		1.37 (1.24 – 1.50)			2.13 (1.44 – 3.14)		1.21 (1.09 – 1.34)		
Low income+university	4.35 (3.16 – 5.98)		1.51 (1.28 – 1.79)			3.96 (2.74 – 5.72)		1.26 (1.05 – 1.53)		
Low income+secondary	3.06 (2.39 – 3.92)		1.57 (1.42 – 1.74)			2.33 (1.75 – 3.10)		1.29 (1.15 – 1.45)		
Low income+primary	4.40 (3.39 – 5.70)		1.84 (1.68 – 2.02)			3.28 (2.42 – 4.45)		1.48 (1.33 – 1.64)		
Toileting (Needs assistance vs. Completely independent)										

SES (ref. high income+university)	1	<0.001	1	<0.001	0.001	1	0.010	1	<0.001	0.064
High income+secondary	1.40 (1.05 – 1.88)		1.03 (0.91 – 1.17)			1.38 (1.00 – 1.91)		0.95 (0.82 – 1.09)		
High income+primary	1.56 (1.09 – 2.24)		1.14 (1.00 – 1.31)			1.38 (0.92 – 2.08)		1.04 (0.89 – 1.21)		
Mid income+university	1.02 (0.55 – 1.91)		1.10 (0.95 – 1.27)			0.71 (0.35 – 1.44)		1.05 (0.89 – 1.24)		
Mid income+secondary	1.74 (1.22 – 2.46)		1.26 (1.12 – 1.41)			1.36 (0.91 – 2.03)		1.11 (0.98 – 1.26)		
Mid income+primary	2.81 (1.85 – 4.25)		1.26 (1.13 – 1.41)			2.01 (1.23 – 3.29)		1.11 (0.98 – 1.26)		
Low income+university	2.56 (1.64 – 3.98)		1.42 (1.16 – 1.73)			1.88 (1.09 – 3.24)		1.28 (1.02 – 1.61)		
Low income+secondary	2.26 (1.62 – 3.14)		1.39 (1.23 – 1.56)			1.31 (0.89 – 1.94)		1.16 (1.01 – 1.33)		
Low income+primary	3.39 (2.42 – 4.75)		1.71 (1.54 – 1.90)			2.00 (1.33 – 3.01)		1.38 (1.22 – 1.56)		
Dressing (Needs assistance vs. Completely independent)										
SES (ref. high income+university)	1	<0.001	1	<0.001	<0.001	1	<0.001	1	<0.001	0.035
High income+secondary	1.22 (0.95 – 1.57)		1.07 (0.95 – 1.20)			1.17 (0.88 – 1.54)		1.00 (0.88 – 1.13)		
High income+primary	1.39 (1.01 – 1.90)		1.12 (0.99 – 1.27)			1.20 (0.84 – 1.71)		1.02 (0.89 – 1.18)		
Mid income+university	1.06 (0.63 – 1.78)		1.14 (1.00 – 1.30)			0.77 (0.43 – 1.39)		1.09 (0.94 – 1.27)		
Mid income+secondary	1.60 (1.18 – 2.16)		1.26 (1.13 – 1.40)			1.23 (0.87 – 1.74)		1.10 (0.98 – 1.24)		
Mid income+primary	2.40 (1.65 – 3.48)		1.27 (1.15 – 1.41)			1.66 (1.07 – 2.59)		1.13 (1.01 – 1.27)		
Low income+university	3.13 (2.17 – 4.50)		1.37 (1.13 – 1.65)			2.04 (1.29 – 3.22)		1.20 (0.97 – 1.48)		
Low income+secondary	2.21 (1.67 – 2.94)		1.38 (1.24 – 1.55)			1.32 (0.95 – 1.85)		1.15 (1.01 – 1.31)		

Low income+primary	3.41 (2.55 – 4.56)		1.72 (1.56 – 1.90)			2.08 (1.46 – 2.95)		1.41 (1.25 – 1.58)		
Low mood (Often/Constantly vs. Never/almost never/Sometimes										
SES (ref. high income+university)	1	<0.001	1	<0.001	<0.001	1	<0.001	1	0.008	<0.001
High income+secondary	1.11 (0.89 – 1.38)		1.01 (0.88 – 1.17)			1.01 (0.80 – 1.27)		0.99 (0.86 – 1.14)		
High income+primary	1.63 (1.24 – 2.14)		0.97 (0.83 – 1.14)			1.37 (1.03 – 1.82)		0.92 (0.78 – 1.08)		
Mid income+university	1.92 (1.34 – 2.75)		1.09 (0.92 – 1.29)			1.78 (1.23 – 2.57)		1.02 (0.86 – 1.22)		
Mid income+secondary	1.78 (1.38 – 2.30)		1.00 (0.87 – 1.14)			1.51 (1.16 – 1.97)		0.90 (0.78 – 1.03)		
Mid income+primary	2.40 (1.71 – 3.37)		1.12 (0.98 – 1.27)			1.96 (1.38 – 2.80)		1.01 (0.88 – 1.15)		
Low income+university	2.86 (2.05 – 3.98)		1.45 (1.16 – 1.82)			2.42 (1.71 – 3.42)		1.35 (1.07 – 1.70)		
Low income+secondary	2.80 (2.20 – 3.56)		1.16 (1.01 – 1.33)			2.22 (1.72 – 2.87)		0.98 (0.85 – 1.14)		
Low income+primary	3.73 (2.88 – 4.82)		1.28 (1.13 – 1.45)			2.80 (2.13 – 3.69)		1.10 (0.96 – 1.26)		
Fatigue (Often/Constantly vs. Never/almost never/Sometimes)										
SES (ref. high income+university)	1	<0.001	1	<0.001	<0.001	1	<0.001	1	<0.001	<0.001
High income+secondary	1.25 (1.09 – 1.43)		0.91 (0.83 – 1.00)			1.19 (1.04 – 1.36)		0.88 (0.81 – 0.97)		
High income+primary	1.29 (1.08 – 1.54)		0.90 (0.81 – 1.00)			1.19 (0.99 – 1.42)		0.84 (0.76 – 0.94)		
Mid income+university	1.64 (1.27 – 2.13)		1.19 (1.07 – 1.33)			1.60 (1.22 – 2.08)		1.19 (1.06 – 1.33)		
Mid income+secondary	1.26 (1.06 – 1.50)		1.07 (0.98 – 1.16)			1.16 (0.97 – 1.38)		0.99 (0.91 – 1.09)		

Mid income+primary	1.55 (1.21 – 1.98)		1.06 (0.97 – 1.15)			1.39 (1.07 – 1.79)		0.99 (0.91 – 1.08)		
Low income+university	1.61 (1.24 – 2.08)		1.31 (1.11 – 1.54)			1.40 (1.07 – 1.83)		1.28 (1.09 – 1.52)		
Low income+secondary	1.59 (1.34 – 1.89)		1.08 (0.98 – 1.19)			1.45 (1.21 – 1.74)		1.01 (0.91 – 1.11)		
Low income+primary	1.90 (1.56 – 2.31)		1.05 (0.96 – 1.14)			1.65 (1.34 – 2.04)		0.95 (0.86 – 1.04)		
Pain (Often/Constantly vs. Never/almost never/Sometimes)										
SES (ref. high income+university)	1	<0.001	1	<0.001	<0.001	1	<0.001	1	<0.001	<0.001
High income+secondary	1.34 (1.14 – 1.59)		1.07 (0.96 – 1.20)			1.28 (1.08 – 1.52)		1.04 (0.93 – 1.17)		
High income+primary	1.54 (1.24 – 1.91)		1.09 (0.97 – 1.24)			1.41 (1.13 – 1.76)		1.05 (0.93 – 1.19)		
Mid income+university	1.51 (1.11 – 2.05)		1.29 (1.13 – 1.48)			1.46 (1.07 – 1.99)		1.25 (1.09 – 1.43)		
Mid income+secondary	2.07 (1.69 – 2.53)		1.29 (1.16 – 1.43)			1.90 (1.55 – 2.34)		1.21 (1.09 – 1.35)		
Mid income+primary	2.44 (1.85 – 3.21)		1.38 (1.25 – 1.53)			2.26 (1.70 – 3.01)		1.31 (1.18 – 1.45)		
Low income+university	2.41 (1.81 – 3.20)		1.54 (1.29 – 1.85)			2.13 (1.59 – 2.87)		1.48 (1.23 – 1.79)		
Low income+secondary	2.52 (2.07 – 3.07)		1.48 (1.32 – 1.65)			2.25 (1.83 – 2.77)		1.35 (1.20 – 1.51)		
Low income+primary	2.43 (1.95 – 3.04)		1.38 (1.25 – 1.53)			2.02 (1.59 – 2.56)		1.28 (1.15 – 1.42)		
General health (Very poor/quite poor vs. very good/quite good)										
SES (ref. high income+university)	1	<0.001	1	<0.001	<0.001	1	<0.001	1	0.011	<0.001
High income+secondary	1.21 (1.01 – 1.45)		1.01 (0.90 – 1.12)			1.13 (0.93 – 1.36)		0.96 (0.86 – 1.08)		

High income+primary	1.28 (1.01 – 1.63)		1.01 (0.89 – 1.14)			1.12 (0.87 – 1.43)		0.93 (0.82 – 1.06)		
Mid income+university	1.39 (0.98 – 1.96)		1.18 (1.03 – 1.35)			1.27 (0.89 – 1.80)		1.15 (1.00 – 1.32)		
Mid income+secondary	1.93 (1.55 – 2.40)		1.15 (1.03 – 1.27)			1.62 (1.29 – 2.04)		1.03 (0.92 – 1.15)		
Mid income+primary	2.79 (2.09 – 3.72)		1.20 (1.08 – 1.32)			2.24 (1.65 – 3.03)		1.08 (0.97 – 1.20)		
Low income+university	2.33 (1.72 – 3.17)		1.52 (1.26 – 1.82)			1.74 (1.26 – 2.42)		1.33 (1.10 – 1.62)		
Low income+secondary	2.28 (1.84 – 2.83)		1.25 (1.12 – 1.40)			1.75 (1.39 – 2.20)		1.07 (0.95 – 1.21)		
Low income+primary	3.12 (2.47 – 3.94)		1.25 (1.13 – 1.39)			2.29 (1.78 – 2.94)		1.06 (0.95 – 1.19)		

[a] P-value from likelihood ratio test of outcome models with and without SES.

[b] P-value from likelihood ratio test of outcome models fitted on the whole study population with and without the SES*age category-interaction.
